# Transvenous Lead Extraction in Patients with Congenital Heart Disease

**DOI:** 10.3390/jcm14124178

**Published:** 2025-06-12

**Authors:** Andrea Csillik, Rita Beata Gagyi, Attila Kardos, Csaba Földesi, Zoltán Som, Mate Vamos, Tamas Szili-Torok

**Affiliations:** 1 Department of Cardiology, Buda Hospital of the Hospitaller Order of St. John of God, 1027 Budapest, Hungary; 2 Doctoral School of Clinical Medicine, University of Szeged, 6720 Szeged, Hungary; 3 Cardiac Electrophysiology Division, Cardiology Centre, Department of Internal Medicine, University of Szeged, 6720 Szeged, Hungaryszili-torok.tamas@med.u-szeged.hu (T.S.-T.); 4 Gottsegen Gyorgy National Cardiovascular Center, 1096 Budapest, Hungary

**Keywords:** congenital heart disease, transvenous lead extraction, TLE, rotational mechanical dilator

## Abstract

**Background/Objectives:** A significant subset of congenital heart disease (CHD) patients undergo a transvenous pacemaker (PM)/implantable cardioverter defibrillator (ICD) lead extraction (TLE) in their lifetime. We aimed to report on the outcome and complexity of TLEs in CHD patients for whom a powered mechanical sheath was used. **Methods**: This retrospective study included 175 consecutive TLEs performed at our centre. Overall, 13 TLEs in CHD patients and 162 TLEs in non-CHD patients were performed. A total of 264 leads were extracted. **Results**: CHD patients were younger than non-CHD patients at the time of their first lead implant (21.2 ± 17 vs. 57.1 ± 18 years; *p* < 0.01) and at the time of lead extraction (33.38 ± 13 vs. 63.31 ± 16 years; *p* < 0.01). The leads extracted from CHD patients were significantly older than the leads extracted from non-CHD patients (median: 8.0 vs. 4.0 years; *p* < 0.01). CHD patients and non-CHD patients did not differ in terms of the procedural (92% vs. 87%; *p* = 0.581) and clinical success rates (100% vs. 91%; *p* = 0.269). The two patient groups did not differ in terms of their procedural complication rate (0% vs. 11%; *p* = 0.191). There were no differences in the extraction techniques used, i.e., rotational mechanical sheaths were used in 61% of CHD extractions and in 38% of non-CHD extractions; *p* = 0.11. **Conclusions:** TLEs that use rotational mechanical sheaths as an advanced technique can be safely and effectively performed in CHD patients. The outcome and complexity of TLEs in CHD patients are comparable with those in non-CHD patients that undergo this procedure.

## 1. Introduction

The overall number of adult congenital heart disease (CHD) patients continues to rise because of significant improvements in medical and surgical approaches over the past few decades, which have improved the survival rates [1]. More than 90% of newborns diagnosed with congenital heart disease reach adulthood [2]. A subset of these patients receive implantable cardiac devices (CIED) in their childhood [3,4]. CIED complications leading to a transvenous pacemaker (PM)/implantable cardioverter defibrillator (ICD) lead extraction (TLE) are not uncommon in this patient population [5,6]. Lead extraction in CHD patients can be technically challenging due to the complex vascular and cardiac anatomy of these patients. Inadvertent patch or baffle injuries may be haemodynamically relevant and electrode remnants may cause thromboembolic complications in CHD patients [7]. Achieving procedural success and avoiding complications are therefore of paramount importance.

Over the past few years, lead extraction centres have gained significant experience in using mechanically powered sheaths [8,9,10,11,12]. The available data regarding the outcome and complexity of lead extractions using only a rotational mechanical dilator as a powered sheath in CHD patients are scarce [13]. The aim of this study was to report on the outcome and complexity of TLEs in CHD patients. We compared the success rate, complication rate, mortality rate, and complexity rate of extractions performed in CHD patients with those of extractions performed in non-CHD patients. We hypothesized that a TLE is a safe and effective procedure for CHD patients, and that the success rate, complication rate, and mortality rate of TLEs performed in CHD patients is similar to TLEs performed in non-CHD patients. Our other hypothesis was that an advanced extraction technique is necessary more often in TLEs performed in CHD patients compared to TLEs performed in non-CHD patients.

## 2. Materials and Methods

### 2.1. Patient Population

In our retrospective study, we included consecutive patients undergoing a transvenous lead extraction procedure at a tertiary referral centre. All TLEs were performed between January 2014 and December 2023 by three expert electrophysiologists at the Gottsegen Gyorgy National Cardiovascular Centre, Budapest, Hungary.

### 2.2. Primary Hypothesis and Study Design

The primary hypothesis of this study was that the success rate, complication rate, and mortality rate of percutaneous lead extractions performed in CHD patients are comparable to the outcomes of TLEs performed in non-CHD patients. We further hypothesized that due to their younger age and unique anatomic characteristics, CHD patients require more advanced extraction techniques than non-CHD patients. In our study, we first defined the success rate, complication rate, mortality rate and procedural complexity rate of TLEs in CHD patients. We then compared these outcome data with those of TLEs performed in non-CHD patients who underwent percutaneous lead extraction during the same time period.

### 2.3. Data Collection

Data from hospital records and chest X-ray images were collected in a database retrospectively. The following demographic data were collected: age, sex, and comorbidities. The indication for lead extraction, the types of implantable cardiac devices, and the lead characteristics, such as the lead implant duration, the number of leads present, the number of leads treated per patient, the number of extracted ICD leads, and the number of extracted abandoned leads, were collected. Methodological data on lead extraction, such as the use of a powered sheath and the procedure’s duration, were also collected. The outcome was characterized by the procedural and clinical success rate, the procedural complication rate, the 30-day mortality rate, and the complexity rate of percutaneous lead extractions. The following procedural complications were recorded: procedure-related death, cardiac avulsion, vascular laceration, valve injury, pericardial effusion, haemothorax, pulmonary embolism, heart failure requiring intervention, haematoma requiring evacuation, and intra-or perioperative blood loss requiring blood transfusion.

### 2.4. Consent and Ethics

The Hungarian national medical ethics committee (the Scientific and Research Ethics Committee of the Scientific Council for Health “ETT TUKEB”) approved the data collection for this study (CHD-01). This study was performed according to the principles of the Declaration of Helsinki.

### 2.5. Definitions

A percutaneous lead extraction, the procedural efficacy, and the complications were defined according to the 2018 EHRA guidelines [14]. Complete procedural success was defined as the removal of all targeted leads and material, with the absence of any permanently disabling complication or procedure-related death [14]. Clinical procedural success was defined as the retention of a small portion of a lead that did not negatively impact the outcome goals of the procedure [14]. A lead extraction was considered simple if only simple manual traction or a locking stylet was required, and was defined as complex if a rotational mechanical sheath or a femoral snare was utilized. The indications for TLEs were categorized as infections (pocket infection, infective endocarditis) or non-infections (lead failure, dislocation, or system upgrade) [15].

### 2.6. Extraction Procedures

TLEs were performed either in the electrophysiology laboratory or in a cardiac surgical operating room equipped with a fluoroscopy system. Local anaesthesia or general anaesthesia was applied based on the heart team’s decision. Standby cardiac surgery was available during all the TLE procedures. Continuous intraoperative transoesophageal echocardiography (TEE) or intracardiac echocardiography (ICE) was added in TLEs when a difficult procedure was anticipated.

First, an incision was made at the site of the pulse generator; next, the leads were disconnected; and then, a dissection was performed along the leads to the suture sleeves. The sleeves were removed and leads were cut. All electrodes were removed using a standard stepwise approach: first, a straight stylet and simple manual traction was applied; then, a locking stylet was used; and last, a mechanically powered sheath (Evolution, Cook Medical, Bloomington, USA, or TightRail, Philips Healthcare, Orlando, FL, USA) was used, if necessary. The rotational mechanical sheath sizes included 9-, 11-, and 13-Fr sheaths. In the case of lead fracture, a femoral approach by applying the snare technique (Needle’s Eye snare (Cook Medical Inc., Bloomington, IN, USA) was also used. A tandem femoral approach was not routinely used during the extraction process. In patients without an infective indication, device re-implantation was performed in the same procedure. In patients with an infective indication, device re-implantation was deferred to an infection-free time point.

### 2.7. Statistical Analysis

The mean and standard deviation (SD) were calculated for normally distributed continuous variables. The median and interquartile range (IQR) were computed for continuous variables with a non-normal distribution. Categorical data were presented as absolute numbers and percentages. To compare the baseline and procedural characteristics between the patient groups, we used an independent *t*-test. To compare non-normally distributed data between patient groups, we used the Mann-Whitney U-test. Survival curves were analysed using a log-rank test. A *p*-value of less than 0.05 was considered significant. The statistical analyses were performed using the JASP software, version 0.16.4 (Intel Corporation, USA).

## 3. Results

### 3.1. Study Population

Our retrospective database of 175 TLEs consisted of 13 extractions performed in 11 CHD patients (2 patients underwent repeated TLEs years after the index procedure) and 162 extractions performed in non-CHD patients.

The baseline patient demographics are presented in Table 1.

The CHD patients were younger than the non-CHD patients at the time of their first lead implant (21.2 ± 17 vs. 57.1 ± 18 years; *p* < 0.01) and at the time of lead extraction (33.4 ± 13 vs. 63.3 ± 16 years; *p* < 0.01). There were more female patients in the CHD group (69%) compared to the non -CHD group (30%); *p* < 0.01.

An ejection fraction of <50% was present in 23% of the CHD patients and in 60% of the non-CHD patients (*p* = 0.06). Among the heart failure patients, there were no significant differences between the two patient groups concerning the heart failure treatment: beta-blocker therapy was used in 66% of the CHD patients and 84% of the non-CHD patients (*p* = 0.418), ACEI/ARB/ARNI therapy was used in 66% of the CHD patients and 74% of the non-CHD patients (*p* = 0.752), and MRA therapy was used regularly in 33% of the CHD patients and 62% of the non-CHD patients (*p* = 0.314).

Prior cardiac surgery was present in 100% of the CHD patients as compared with 13% of the non-CHD patients (*p* < 0.01). A prosthetic heart valve was present in a significantly greater number of the CHD patients (30.7%) compared to the non-CHD patients (7.3%) (*p* = 0.005). Diabetic patients were present in a similar proportion in both groups (7.7% of the CHD patients and 27.75% of the non-CHD patients; *p* = 0.113.

A summary of the underlying heart disease of the CHD patients is shown in Table 1. Detailed demographics of the individual CHD extractions are given in Table 2.

Cardiac abnormalities were mostly moderate, as classified by the European Society of Cardiology’s CHD complexity scheme [16]. In five cases, the underlying heart disease was severe, and in one case, the cardiac abnormality was mild. The most common underlying cardiac abnormality was the coarctation of the aorta (n:4). All of these aortic coarctations were repaired with an end-to-end anastomosis in childhood, and one patient subsequently underwent a Bentall procedure in early adulthood. Tetralogy of Fallot (n:3) with complete surgical reconstruction, including pulmonary homograft implantation, was the second most common underlying heart disease, followed by the d-transposition of the great arteries, all the patients of which had previously undergone a Senning operation (n:3). Two patients had a double outlet right ventricle palliated surgically with a Fontan circulation and one patient had a ventricular septal defect repaired through patch closure. In seven cases, an additional cardiac abnormality was present, among which an atrial or ventricular septal defect was the most common pathology. In two cases, an incomplete AV septal defect and a cleft mitral valve were present, and one patient had a persistent left superior vena cava.An extracardiac abnormality was present in one case, where the coarctation of the aorta was accompanied by Turner syndrome.

**Figure 1:**
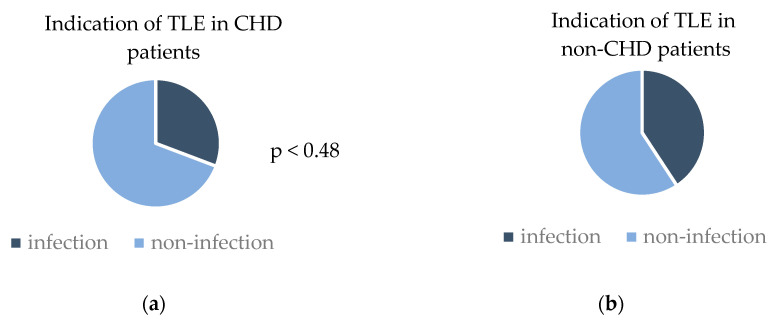
Indication for transvenous lead extraction (TLE) in (**a**) CHD patients and (**b**) non-CHD patients. (**a**) Rates of infectious and non-infectious indications in congenital heart disease (CHD) patients; (**b**) rates of infectious and non-infectious indications in non-congenital heart disease (non-CHD) patients.

The patients’ indications for lead extractions was a system infection in 31% of the CHD patients and 41% of the non-CHD patients (*p* = 0.48). The indications for a TLE are provided in Figure 1.

**Figure 2:**
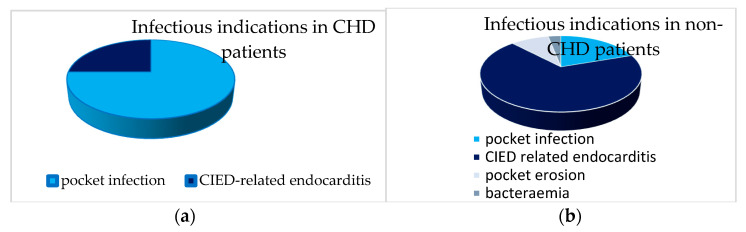
Infectious indications for transvenous lead extraction (TLE) in (**a**) CHD patients and (**b**) non-CHD patients. (**a**) Rates of an isolated pocket infection, cardiac implantable electronic device (CIED)-related endocarditis, isolated pocket erosion, and bacteraemia in congenital heart disease (CHD) patients; (**b**) rates of isolated pocket infections, CIED-related endocarditis, isolated pocket erosion, and bacteraemia in non-congenital heart disease (non-CHD) patients.

Infectious indications were further subclassified into the following categories: an isolated pocket infection, isolated pocket erosion, cardiac implantable electronic device (CIED)-related endocarditis, and bacteraemia. In the CHD patients, 75% of infectious TLEs were performed because of an isolated pocket infection, and only 25% of infectious TLEs were performed because of CIED-related endocarditis, whereas in the non-CHD patients, most infectious TLEs were performed with an indication of CIED-related endocarditis (68.1%). The subclassification of infectious indications in the CHD patients and non-CHD patients is provided in Figure 2.

### 3.2. Device Type and Lead Characteristics

The device types present at the time of extraction and the lead characteristics are shown in Table 3.

Most CHD patients had a dual chamber pacemaker at the time of the extraction (61.5%). The most common devices in the non-CHD group were CRT-Ds (27.7%) and dual chamber pacemakers (27.7%).

Overall, 264 leads were extracted, 22 leads were removed from the CHD patients and 242 leads were extracted from the non-CHD patients. The median lead dwelling time was higher for the CHD patients (8 years) than for non-CHD patients (4 years) (*p* < 0.01). The majority of the CHD patients and non-CHD patients had two leads present at the time of extraction (61% vs. 35%; *p* = 0.448). One lead was extracted in most of the CHD (54%) and non-CHD (67%) patients (*p* = 0.594). The rate of ICD lead extraction was numerically lower in the CHD patients compared to the non-CHD patients; however, this difference was statistically not significant (15% vs. 40%; *p* = 0.07). Abandoned leads were extracted more often from CHD patients (23%) than from non-CHD patients (6%) (*p* = 0.025).

### 3.3. TLE Outcomes in CHD Patients: Success Rate, Complication Rate, Survival and Rate of Advanced Technique Use

The complete procedural success rate in the CHD patients was 92%, and the clinical procedural success rate among the CHD was 100%. Neither minor nor major procedural complications were observed in the CHD group. The procedure-related mortality was 0% in the CHD patients. The 30-day mortality rate was 8.3% among the CHD patients: one patient was lost to follow-up, eleven out of twelve (91.7%) patients survived, and one patient (8.3%) died of septic shock following the procedure. Percutaneous extractions in the CHD patients required the use of rotational mechanical sheaths and/or a femoral snare in 61% of cases. The procedural outcome data are summarized in Table 3.

### 3.4. Comparison of TLE Outcomes in CHD Patients with Non-CHD Patients

There were no differences between the CHD patients and the non-CHD patients in the procedural success rate (92% vs. 87%; *p* = 0.581) or the clinical success rate (100% vs. 91%; *p* = 0.269). The two patient groups did not differ in their procedural complication rate either (0% vs. 11%; *p* = 0.191).

**Figure 3:**
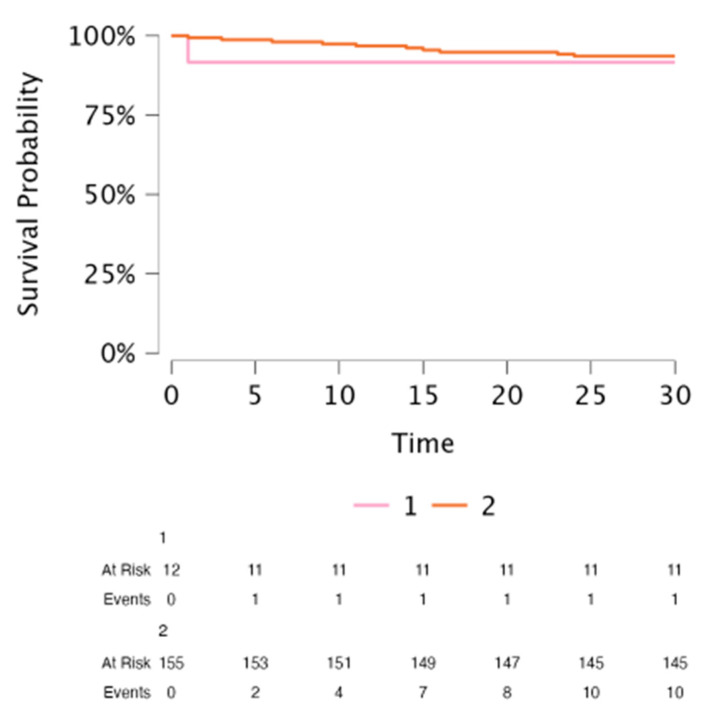
The 30-day survival in CHD patients and non-CHD patients. CHD: Congenital Heart Disease.

The procedure-related mortality was 0% in both groups. 30-day mortality rate was not different between the CHD (8.3%) and non-CHD (6.6%) patients (*p* = 0.825); the cause of death was septic shock for all patients. The 30-day survival in the CHD patients and the non-CHD patients is presented in Figure 3.

There were no differences in the TLE techniques utilized. Simple manual traction was used in 30% of the CHD extractions and 48% of the non-CHD extractions (*p* = 0.21) and it had an overall clinical success rate of 97.5% and an overall procedural success rate of 96.3%. A locking stylet and manual traction was applied in 8.3% of the CHD extractions and 11% of the non-CHD extractions (*p* = 0.641). A complex technique was necessary in 61% (8 of 13) of the CHD extractions, and in 38% (63 of 162) of the non-CHD extractions (*p* = 0.11). The distribution of Evolution and TightRail use was similar in the two patient groups: Evolution powered sheaths were used in 23% of CHD extractions and 10% of non-CHD extractions, TightRail powered sheaths were applied in 15% of the CHD extractions and 22% of the non-CHD extractions (*p* = 0.191). However, the femoral snare was more often necessary as bail-out technique in the CHD patients (23%) compared to the non-CHD patients (4.9%) (*p* = 0.01).

## 4. Discussion

The main finding of the present study was that, when using a stepwise approach with a mechanically powered sheath as the last step, a percutaneous lead extraction in surgically corrected CHD patients can be accomplished with a high efficacy and safety, comparable with TLEs performed in non-CHD patients.

### 4.1. TLEs in Surgically Repaired CHD Patients

Surgically corrected congenital heart disease patients represent a unique patient population, in that they pose special technical challenges to TLEs. The difficulties inherent to the use of TLEs in surgically repaired CHD patients include the younger age of the patients, a longer lead implant duration, and cardiac abnormalities arising not only from congenital malformations, but also from the postoperative conditions. Previous reports have shown that patients with a greater anatomic complexity have an increased risk of developing a lead complication; thus, a transvenous PM/ICD lead extraction becomes a necessity in a greater proportion of postoperative CHD patients [17]. Therefore, understanding the outcome of a percutaneous lead extraction in this special postoperative patient group is essential.

Previous reports of lead extractions in CHD patients that focused on a mixed population of CHD patients with and without a previous surgical correction have shown that a percutaneous lead extraction is effective and relatively safe in this patient group [18]. However, there are limited data on the safety and efficacy of TLEs performed in surgically corrected CHD patients. Our study, in which all the CHD patients were surgically reconstructed, suggests that percutaneous lead extraction can be performed safely and effectively in this patient group, despite their unique anatomic characteristics.

### 4.2. Use of Mechanically Powered Sheaths in CHD Patients: Efficacy

Concerning the technique of TLEs in CHD patients, previous studies have shown that laser-powered lead extractions [18,19,20,21] and radiofrequency-powered sheath use [22] have a clinical success rate of 74–94%. Mechanically powered sheath use has a well-established efficacy profile; it is a safe and feasible procedure in the general population, even with same-day-discharge in selected patients [23]. Over the past decade, extraction centres have gained significant experience in using this technique of percutaneous lead extraction [8,9,10,11,12,23]. However, little is known about the efficacy of mechanically powered sheath use in surgically corrected CHD patients. In our study, all the extractions were performed using mechanically powered sheaths as advanced extraction tools. We report that, in surgically corrected CHD patients, lead extractions had a clinical success rate of 100%, an efficacy rate that exceeds the previously reported efficacy rate of laser-or radiofrequency-powered extractions in CHD patients. The superior performance of mechanically powered sheath use in CHD patients compared to laser- or radiofrequency-powered extractions might be explained by the observation that younger patients—such as CHD patients—form excessive calcific adhesions around intracardiac leads [24], and this calcific mass might be better destroyed using a rotational mechanical TLE rather than laser-powered sheaths.

### 4.3. Use of Mechanically Powered Sheaths in CHD Patients: Safety

Former reports on the complication rate of laser-powered TLEs in CHD patients have shown a complication rate of 5.5–17% [13,19,21,25]. In the current report, despite the difficulties inherent in TLEs performed on CHD patients, no procedural complications occurred during the TLEs in surgically corrected CHD patients. We presume that the previous cardiac surgery had a protective role in reducing the risk of pericardial effusion due to postoperative pericardial adhesion.

### 4.4. Abandoned Leads

Defining the risk of extracting abandoned leads is particularly important in surgically repaired CHD patients. Previous studies have shown that the extraction of abandoned leads increases the complication rate and decreases the success rate of TLEs [8,26]. However, abandonment has also been shown to carry the risk of infection, future lead-to-lead interactions, tricuspid regurgitation and venous occlusion [27,28], an issue which is of paramount importance in CHD patients repaired with baffles. It is well known that the policy to abandon a non-functioning lead often increases the difficulty of a future extraction [29]; thus, the benefit of extracting a non-functioning lead could be much greater in younger patients. In our single-centre study, where abandoned leads were present in 23% of the CHD patients, the extraction of abandoned leads did not increase the complication rate of the TLEs. According to the current guidelines, the extraction of non-functional leads that have no negative arrhythmic or thromboembolic impact is a class IIa indication or—when the aim is to facilitate magnetic resonance imaging—a class IIb indication [30]. In everyday practice, this means that in such situations, a shared decision-making process about extraction is advised. Our finding that abandoned leads can be safely extracted in CHD patients helps to refine such shared decisions, and argues for the extraction of non-functioning leads in young CHD patients after a careful risk-benefit analysis.

### 4.5. Study Limitations

The relatively small sample size of the CHD group is the most important limitation of our analysis; however, it should be noted that surgically repaired CHD patients undergoing a percutaneous lead extraction is a unique patient population. This study represents a single-centre experience; however, as our lead extraction centre is the only centre dedicated to CHD percutaneous extractions in our country, it was not possible to include patients from other centres. Continuous intraoperative TEE or ICE monitoring was performed in only 10.9% of the TLEs, which is another limitation of our study [31]. Since intraoperative imaging data were not consistently available, we did not include it in our retrospective analysis. Another limitation of our study is that, although it was common practice among every operator to use a stepwise approach in TLE procedures, operator discretion could not be excluded in the actual steps of the TLE. The procedure duration was collected as skin-to skin time, which indicated the duration of the extraction procedure itself only in infectious indications, where a new electrode was not simultaneously implanted. Although the procedure duration is a relevant issue, it was not statistically interpretable for the above reason.

## 5. Conclusions

In conclusion, in surgically corrected CHD patients, a percutaneous lead extraction can be performed safely and effectively, and is comparable to extractions performed in patients without CHD. A complex technique was not required more often in CHD patients than it is for non-CHD patients. Our findings suggest that this approach to remove non-functioning leads seems justified, even in CHD patients, after a careful risk-benefit analysis.

## Figures and Tables

**Table 1:** Baseline demographics.

	CHD (*n* = 13)	Non-CHD (*n* = 162)	*p*-Value
Patient age at time of lead implantation (years)	21.2 ± 17	57.1 ± 18	<0.01
Patient age at time of extraction (years)	33.4 ± 13	63.3 ± 16	<0.01
Female	9 (69%)	49 (30%)	<0.01
HFrEF and HFmrEF	3 (23%)	97 (60%)	0.06
Beta-blocker therapy	2 (66%)	80 (84%)	0.418
ACEI/ARB/ARNI therapy	2 (66%)	71 (74%)	0.752
MRA therapy	1 (33%)	59 (62%)	0.314
Prior cardiac surgery	13 (100%)	21 (13%)	<0.01
Previous valve prothesis implantation	4 (30.7%)	12 (7.4%)	<0.01
Diabetes mellitus	1 (7.7%)	45 (27.75)	0.113
		*n*	
Underlying CHD heart disease	d-Transposition of the great arteries St.p. op. Senning	3 (23%)	
	Double outlet right ventricle St.p. op. Fontan	2 (15%)	
	Tetralogy of Fallot	3 (23%)	
	Coarctation of the aorta	4 (30%)	
	Ventricular septal defect	1 (7%)	

ACEI: Angiotensin-Converting Enzyme Inhibitors, ARB: Angiotensin Receptor Blocker, ARNI: Angiotensin Receptor -Neprilysin Inhibitor, CHD: Congenital Heart Disease G. HFrEF: Heart Failure with Reduced Ejection Fraction, HFmrEF: Heart Failure with Mildly Reduced Ejection Fraction, MRA: Mineralocorticoid Receptor Antagonist, *n*: Number.

**Table 2:** Detailed demographics of individual CHD extractions.

Extraction No	Age	Sex	EF %	CHD Complexity Classification	Basic CHD	Additional Cardiac Abnormality	Extracardiac Abnormality	Cardiac Repair Surgery	Number of Extracted Leads	Implant Duration of Oldest Extracted Lead (years)	RV Diam. (mm)
1	24	male	55	mild	ventricular septal defect	none	none	patch repair	3	23	24
2	14	female	73	moderate	coarctation of the aorta	incomplete AV septal defect and cleft mitral valve	none	extended end-to-end anastomosis	1	6	31
3	41	female	51	moderate	tetralogy of Fallot	none	none	complete reconstruction	2	6	40
4	35	male	31	severe	d-transposition of the great arteries	none	none	Senning operation	1	6	54
5	39	female	55	severe	double outlet right ventricle	ASD, pulmonary stenosis	none	Fontan operation	1	23	31
6	27	male	50	severe	d-transposition of the great arteries	ASD	none	Senning operation	3	25	42
7	16	female	69	moderate	coarctation of the aorta	incomplete AV septal defect and cleft mitral valve	none	end-to-end anastomosis	2	8	31
8	57	female	63	moderate	tetralogy of Fallot	none	none	complete reconstruction	2	16	46
9	24	female	74	moderate	coarctation of the aorta	VSD, ASD	none	end-to-end anastomosis, ASD and VSD patch repair	1	14	29
10	48	female	27	moderate	coarctation of the aorta	persistent vena cava sup.sin.	Turner syndrome	Bentall operation	1	1.4	31
11	37	male	30	severe	d-transposition of the great arteries	none	none	Senning operation	1	6	30
12	24	female	65	severe	double outlet right ventricle	VSD, pulmonary stenosis	none	Fontan operation	3	21	41
13	48	female	64	moderate	tetralogy of Fallot	none	none	complete reconstruction	1	1	51

ASD: Atrial Septal Defect, AV: Atrioventricular, CHD: Congenital Heart Disease, VSD: Ventricular Septal Defect.

**Table 3:** Extracted device types, lead characteristics, and outcomes.

Devices:	CHD Patients (*n* = 13)	Non-CHD Patients (*n* = 162)	*p*-Value
Single chamber (atrial) PM	1 (7.7%)	2 (1.23%)	0.004
Single chamber (ventricular) PM	1 (7.7%)	10 (6.17%)	
Dual chamber PM	8 (61.5%)	45 (27.7%)	
Single chamber VDD PM	0	3 (1.85%)	
CRT-P	0	16 (9.9%)	
Single chamber ICD	0	26 (16%)	
Single chamber VDD ICD	0	8 (4.9%)	
Dual chamber ICD	3 (23%)	7 (4.3%)	
CRT-D	0	45 (27.7%)	
**Leads:**	**CHD patients (*n* = 22)**	**Non-CHD patients (*n* = 242)**	***p*-value**
Lead age at time of extraction (median) (years)	8	4	<0.01
Lead age: min max (years)	1.4–25	1–28	
Number of leads present per patient			0.448
1 lead	2 (15%)	44 (27%)	
2 leads	8 (61%)	58 (35%)	
3 leads	3 (23%)	52 (32%)	
4 leads	0	6 (3.7%)	
5 leads	0	2 (1.2%)	
Number of leads treated per patient			0.594
1 lead	7 (54%)	109 (67%)	
2 leads	3 (23%)	31 (19%)	
3 leads	3 (23%)	19 (12%)	
4 leads	0	3 (2%)	
Number of ICD lead extractions	2 (15%)	65 (40%)	0.07
Number of abandoned lead extractions	3 (23%)	10 (6%)	0.025
**Outcomes:**	**CHD patients** **(*n* = 13)**	**Non-CHD patients (*n* = 162)**	***p*-value**
Complete procedural success	12 (92%)	140 (87%)	0.581
Clinical success	13 (100%)	148 (91%)	0.269
Procedural complications	0	19 (11%)	0.191
30-day mortality	1 (8.3%)	10 (6.6%)	0.825
Use of simple manual traction	4 (30%)	79 (48%)	0.211
Use of locking stylet and manual traction	1 (8.3%)	18 (11%)	0.641
Use of powered sheath	8 (61%)	63 (38%)	0.110
use of Evolution powered sheath	3 (23%)	17 (10%)	0.191
use of TightRail powered sheath	2 (15%)	37 (22%)	
use of femoral snare	3 (23%)	8 (4.9%)	0.01

CHD: Congenital Heart Disease, CRT-P: cardiac resynchronisation therapy-pacemaker, CRT-D: cardiac resynchronisation therapy-defibrillator, ICD: implantable cardioverter defibrillator, PM: Pacemaker, VDD: ventricular lead with atrial sensing.

## Data Availability

Readers can access the data supporting the conclusions of this study from the author upon request.

## Abbreviations

The following abbreviations are used in this manuscript:

CHD	Congenital heart disease
ICD	Implantable cardioverter defibrillator
PM	Pacemaker
TLE	Transvenous lead extraction

## References

[1] Van Der Linde D., Konings E.E., Slager M.A., Witsenburg M., Helbing W.A., Takkenberg J.J., Roos-Hesselink J.W. (2011). Birth prevalence of congenital heart disease worldwide: A systematic review and meta-analysis. J. Am. Coll. Cardiol..

[2] Khairy P., Ionescu-Ittu R., Mackie A.S., Abrahamowicz M., Pilote L., Marelli A.J. (2010). Changing mortality in congenital heart disease. J. Am. Coll. Cardiol..

[3] Khairy P., Van Hare G.F., Balaji S., Berul C.I., Cecchin F., Cohen M.I., Daniels C.J., Deal B.J., Dearani J.A., de Groot N. (2014). PACES/HRS expert consensus statement on the recognition and management of arrhyth mias in adult congenital heart disease: Developed in partnership between the Pediatric and Congenital Electrophysiology devel oped in partnership between the Pediatric and Congenital Electrophysiology Society (PACES) and the Heart Rhythm Society (HRS). Endorsed by the governing bodies of PACES, HRS, the American College of Cardiology (ACC), the American Heart Association (AHA), the European Heart Rhythm Association (EHRA), the Canadian Heart Rhythm Society (CHRS), and the International Society for Adult Congenital Heart Disease (ISACHD). Can. J. Cardiol..

[4] Koyak Z., Harris L., de Groot J.R., Silversides C.K., Oechslin E.N., Bouma B.J., Budts W., Zwinderman A.H., Van Gelder I.C., Mulder B.J.M. (2012). Sudden cardiac death in adult congenital heart disease. Circulation.

[5] Czosek R.J., Meganathan K., Anderson J.B., Knilans T.K., Marino B.S., Heaton P.C. (2012). Cardiac rhythm devices in the pediatric population: Utilization and complications. Heart Rhythm.

[6] Wilkoff B.L., Love C.J., Byrd C.L., Bongiorni M.G., Carrillo R.G., Crossley G.H., Epstein L.M., Friedman R.A., Kennergren C.E., Mitkowski P. (2009). Transvenous lead extraction: Heart Rhythm Society expert consensus on facilities, training, indications, and patient management. Heart Rhythm.

[7] Khairy P., Landzberg M.J., Gatzoulis M.A., Mercier L.A., Fernandes S.M., Côté J.M., Lavoie J.P., Fournier A., Guerra P.G., Frogoudaki A. (2006). Epicardial Versus Endocardial pacing and Thromboembolic events Investigators. Transvenous pacing leads and systemic thromboemboli in patients with intracardiac shunts: A multicenter study. Circulation.

[8] Bongiorni M.G., Kennergren C., Butter C., Deharo J.C., Kutarski A., Rinaldi C., Romano S.L., Maggioni A.P., Andarala M., Auricchio A. (2017). The European Lead Extraction ConTRolled (ELECTRa) study: A European Heart Rhythm Association (EHRA) Registry of Transvenous Lead Extraction Outcomes. Eur. Heart J..

[9] Starck C.T., Gonzalez E., Al-Razzo O., Mazzone P., Delnoy P.-P., Breitenstein A., Steffel J., Eulert-Grehn J., Lanmüller P., Melillo F. (2020). Results of the Patient-Related Outcomes of Mechanical lead Extraction Techniques (PROMET) study: A multicentre retrospective study on advanced mechanical lead extraction techniques. Europace.

[10] Sharma S., Lee B.K., Garg A., Peyton R., Schuler B.T., Mason P., Delnoy P.P., Gallagher M.M., Hariharan R., Schaerf R. (2021). Performance and outcomes of transvenous rotational lead extraction: Results from a prospective, monitored, international clinical study. Heart Rhythm O2.

[11] Zsigmond E.-J., Saghy L., Benak A., Miklos M., Makai A., Hegedus Z., Alacs E., Agocs S., Vamos M. (2023). A head-to-head comparison of laser vs. powered mechanical sheaths as first choice and second line extraction tools. Europace.

[12] Yap S.-C., Bhagwandien R.E., Theuns D.A.M.J., Yasar Y.E., de Heide J., Hoogendijk M.G., Kik C., Szili-Torok T. (2021). Efficacy and safety of transvenous lead extraction using a liberal combined superior and femoral approach. J. Interv. Card. Electrophysiol..

[13] Pham T.D.N., Cecchin F., O’Leary E., Fynn-Thompson F., Triedman J.K., Gauvreau K., Mah D.Y. (2022). Lead Extraction at a Pediatric/Congenital Heart Disease Center: The Importance of Patient Age at Implant. JACC Clin. Electrophysiol..

[14] Bongiorni M.G., Burri H., Deharo J.C., Starck C., Kennergren C., Saghy L., Rao A., Tascini C., Lever N., Kutarski A. (2018). 2018 EHRA expert consensus statement on lead extraction: Recommendations on definitions, endpoints, research trial design, and data collection requirements for clinical scientific studies and registries: Endorsed by APHRS/HRS/LAHRS. Europace.

[15] Blomström-Lundqvist C., Traykov V., Erba P.A., Burri H., Nielsen J.C., Bongiorni M.G., Poole J., Boriani G., Costa R., Deharo J.-C. (2020). European Heart Rhythm Association (EHRA) international consensus document on how to prevent, diagnose, and treat cardiac implantable electronic device infections-endorsed by the Heart Rhythm Society (HRS), the Asia Pacific Heart Rhythm Society (APHRS), the Latin American Heart Rhythm Society (LAHRS), International Society for Cardiovascular Infectious Diseases (ISCVID), and the European Society of Clinical Microbiology and Infectious Diseases (ESCMID) in collaboration with the European Association for Cardio-Thoracic Surgery (EACTS). Eur. Heart J..

[16] Baumgartner H., De Backer J., Babu-Narayan S.V., Budts W., Chessa M., Diller G.-P., Lung B., Kluin J., Lang I.M., Meijboom F. (2021). 2020 ESC Guidelines for the management of adult congenital heart disease. Eur. Heart J..

[17] Albertini L., Kawada S., Nair K., Harris L. (2023). Incidence and Clinical Predictors of Early and Late Complications of Implantable Cardioverter-Defibrillators in Adults With Congenital Heart Disease. Can. J. Cardiol..

[18] Fender E.A., Killu A.M., Cannon B.C., Friedman P.A., Mcleod C.J., Hodge D.O., Broberg C.S., Henrikson C.A., Cha Y.-M. (2017). Lead extraction outcomes in patients with congenital heart disease. Europace.

[19] Khairy P., Roux J., Dubuc M., Thibault B., Guerra P.G., Macle L., Mercier L., Dore A., Roy D., Talajic M. (2007). Laser lead extraction in adult congenital heart disease. J. Cardiovasc. Electrophysiol..

[20] McCanta A.C., Kong M.H., Carboni M.P., Greenfield R.A., Hranitzky P.M., Kanter R.J. (2013). Laser lead extraction in congenital heart disease: A case-controlled study. Pacing Clin. Electrophysiol..

[21] Gourraud J.B., Chaix M.A., Shohoudi A., Pagé P., Dubuc M., Thibault B., Poirier N.C., Dore A., Marcotte F., Mongeon F.-P. (2018). Transvenous Lead Extraction in Adults With Congenital Heart Disease: Insights From a 20-Year Single-Center Experience. Circ. Arrhythm. Electrophysiol..

[22] Cecchin F., Atallah J., Walsh E.P., Triedman J.K., Alexander M.E., Berul C.I. (2010). Lead extraction in pediatric and congenital heart disease patients. Circ. Arrhythm. Electrophysiol..

[23] Gianni C., Elchouemi M., Helmy R., Spinetta L., La Fazia V.M., Pierucci N., Asfour I., Della Rocca D.G., Mohanty S., Bassiouny M.A. (2024). Safety and feasibility of same-day discharge following uncomplicated transvenous lead extraction. J. Cardiovasc. Electrophysiol..

[24] Esposito M., Kennergren C., Holmström N., Nilsson S., Eckerdal J., Thomsen P. (2002). Morphologic and immunohistochemical observations of tissues surrounding retrieved transvenous pacemaker leads. J. Biomed. Mater. Res..

[25] Moak J.P., Freedenberg V., Ramwell C., Skeete A. (2000). Effectiveness of excimer laser-assisted pacing and ICD lead extraction in children and young adults. Pacing Clin. Electrophysiol..

[26] Hussein A.A., Tarakji K.G., Martin D.O., Gadre A., Fraser T., Kim A., Brunner M.P., Barakat A.F., Saliba W.I., Kanj M. (2017). Cardiac Implantable Electronic Device Infections: Added Complexity and Suboptimal Outcomes With Previously Abandoned Leads. JACC Clin. Electrophysiol..

[27] Kolodzinska K., Kutarski A., Grabowski M., Jarzyna I., Małecka B., Opolski G. (2012). Abrasions of the outer silicone insulation of endocardial leads in their intracardiac part: A new mechanism of lead-dependent endocarditis. Europace.

[28] Issa Z. An Approach to Transvenous Lead Extraction in Patients with Malfunctioning or Superfluous Leads EP LabDigest 2022. https://www.hmpgloballearningnetwork.com/site/eplab/approach-transvenous-lead-extraction-patients-malfunctioning-or-superfluous-leads.

[29] Segreti L., Rinaldi C.A., Claridge S., Svendsen J.H., Blomstrom-Lundqvist C., Auricchio A., Butter C., Dagres N., Deharo J.-C., Maggioni A.P. (2019). Procedural outcomes associated with transvenous lead extraction in patients with abandoned leads: An ESC-EHRA ELECTRa (European Lead Extraction ConTRolled) Registry Sub-Analysis. Europace.

[30] Kusumoto F.M., Schoenfeld M.H., Wilkoff B.L., Berul C.I., Birgersdotter-Green U.M., Carrillo R., Cha Y.-M., Clancy J., Deharo J.-C., Ellenbogen K.A. (2017). 2017 HRS expert consensus statement on cardiovascular implantable electronic device lead management and extraction. Heart Rhythm.

[31] Strachinaru M., Kievit C.M., Yap S.C., Hirsch A., Geleijnse M.L., Szili-Torok T. (2019). Multiplane/3D transesophageal echocardiography monitoring to improve the safety and outcome of complex transvenous lead extractions. Echocardiography.

